# Effect of Short-Course Oral Ciprofloxacin on Isoflavone Pharmacokinetics following Soy Milk Ingestion in Healthy Postmenopausal Women

**DOI:** 10.1155/2019/7192326

**Published:** 2019-04-11

**Authors:** Nathathai Temyingyong, Nut Koonrungsesomboon, Nutthiya Hanprasertpong, Mingkwan Na Takuathung, Supanimit Teekachunhatean

**Affiliations:** ^1^Graduate School, Chiang Mai University, Chiang Mai 50200, Thailand; ^2^Department of Pharmacology, Faculty of Medicine, Chiang Mai University, Chiang Mai 50200, Thailand; ^3^Musculoskeletal Science and Translational Research (MSTR), Chiang Mai University, Chiang Mai 50200, Thailand; ^4^Center of Thai Traditional and Complementary Medicine, Faculty of Medicine, Chiang Mai University, Chiang Mai 50200, Thailand

## Abstract

Soy isoflavones have several potential benefits related to postmenopausal health. Isoflavone glycosides, found predominantly in nonfermented soy products, e.g., soy milk, require conversion by gut microbiota to their respective bioavailable aglycones prior to absorption into portal circulation. Use of short-course oral ciprofloxacin for the treatment of acute uncomplicated cystitis, the incidence of which is increasing among postmenopausal women, might adversely affect gut microbiota. The objective of this one-group pre-post treatment study was to determine the effect of short-course oral ciprofloxacin on isoflavone pharmacokinetics in healthy postmenopausal women. Eleven postmenopausal subjects were assigned to consume a single oral dose of 375 mL UHT soy milk (SOY phase). Blood samples were collected immediately before soy milk ingestion and at specific times for 32 hours after soy milk ingestion. Following a washout period of at least seven days, subjects were assigned to take 250 mg oral ciprofloxacin after breakfast and dinner for three days, followed by a single oral dose of 375 mL UHT soy milk the next day (CIPRO/SOY phase). Blood samples were collected at the same time points as in the SOY phase. Plasma samples were treated with *β*-glucuronidase/sulfatase and plasma concentrations of aglycones (genistein and daidzein) were determined using high-performance liquid chromatography. C_max_, AUC_0-t_, and AUC_0-∞_ of both aglycones and T_max_ of genistein obtained from the CIPRO/SOY phase were significantly lower than those obtained from the SOY phase, while T_max_ of daidzein and t_1/2_ of both aglycones in the two phases were not significantly different.

## 1. Introduction

Soy isoflavones, nonsteroidal polyphenolic compounds found in soybeans [1], are structurally similar to 17*β*-estradiol and have estrogen-like effects [2, 3]. Evidence suggests that soy isoflavones have several potential benefits related to women's health, such as relief of postmenopausal vasomotor symptoms [4] as well as prevention of estrogen-related cancer [5–7], cardiovascular disease [8, 9], and osteoporosis [10, 11].

Soy isoflavones occur in three aglycone structures (daidzein, genistein, and glycitein), which can enter into three *β*-glycoside conjugates (daidzin, genistin, and glycitin), each with its corresponding acetyl- and malonyl-glycoside conjugates. As glycitein and its glycoside conjugates account for less than 5-10% of the total isoflavones in soy-based products, most studies have focused on daidzein and genistein and their respective glycoside conjugates [12].

The popularity of soy milk consumption is increasing worldwide because soy milk is an important beverage source of isoflavones [13]. It is also the preferred alternative to cow's milk for individuals with lactose intolerance [13]. Regular consumption of isoflavone-rich soy milk alleviates climacteric symptoms (both somatic and urogenital domain symptoms) in peri- and postmenopausal women [14] and helps prevent lumbar spine bone loss in postmenopausal women [15].

Genistin and daidzin, both *β*-glycoside conjugates, have been found to be the main isoflavone components in soy milk [13, 16]. Glycosides are poorly absorbed in the gastrointestinal tract, requiring gut microbiota-mediated conversion to aglycone forms prior to absorption into portal circulation [17, 18]. Thus gut microbiota plays a crucial role in isoflavone absorption and may contribute significantly to the health benefits of isoflavones [19].

Short-course ciprofloxacin (a broad-spectrum fluoroquinolone antibiotic) is one of the recommended antibiotic regimens for treatment of acute uncomplicated cystitis [20], the incidence of which is increasing among postmenopausal women [19]. However, due to its broad spectrum effect against both gram-negative and gram-positive microorganisms, oral ciprofloxacin may also affect the human gut microbiota, resulting in altered isoflavone pharmacokinetics, in particular, a reduction of isoflavone absorption [21]. For that reason, it is postulated that food-drug interaction might occur in postmenopausal women with acute uncomplicated cystitis following coadministration of soy-based products and oral ciprofloxacin. This study aimed to evaluate the effect of short-course oral ciprofloxacin on the pharmacokinetics of soy isoflavones in healthy postmenopausal women.

## 2. Materials and Methods

### 2.1. Study Design

This study was a one-group pre-post treatment study. The study was approved by the Human Research Ethics Committee, Faculty of Medicine, Chiang Mai University, and complied with the Declaration of Helsinki. This trial was registered with Thai Clinical Trials Registry (TCTR): TCTR20180118003.

### 2.2. Subjects

The number of subjects enrolled in this study was determined by the sample size calculation for testing two dependent means (two-tailed test) [22, 23] using the following equation, where *σ* is the standard deviation (SD), delta (∆) is the difference between the two phases, alpha (*α*) is the significance level, and beta (*β*) is the type II error probability.

(1) $$\begin{array}{c} n=\frac{{\left(z_{1-\alpha /2}+z_{1-\beta }\right)}^{2}\sigma^{2}}{{∆}^{2}} \end{array}$$

In this study, the extent of absorbed soy isoflavone genistein (consistent with the area under the concentration-time curve, AUC) was the main criteria for comparison of isoflavone bioavailability. The mean difference in AUC (∆) between pre- and posttreatment was estimated to be 4,800 ng.h/mL and the SD of AUC (*σ*) was assumed to be 5,500. The required sample size to achieved 80% power (*β* = 0.2) at *α* = 0.05 was at least 11 subjects.

Eleven Thai postmenopausal women, aged more than 45 years with a postmenopausal status of more than one year and serum follicle-stimulating hormone (FSH) concentration of greater than 40 IU/L, were enrolled in this study. Their body mass index (BMI) was between 18 and 25 kg/m^2^. All subjects were in good health based on their medical history and a physical examination. Routine blood examination (10 mL from each subject), including complete blood count as well as kidney (BUN, creatinine) and liver function tests, was carried out to identify and exclude subjects with hematological diseases or impaired kidney/liver function. Subjects with known contraindications or hypersensitivity to soy isoflavones or ciprofloxacin were excluded as were women with a history of breast disease, malignancy, cardiovascular or pulmonary disorders, or musculoskeletal disease, e.g., neck or chest pain, back pain, achiness, arthralgia, joint stiffness, or flare-up of gout. Other exclusion criteria included a history of regular consumption of alcohol-containing beverages, use of antibiotics or laxatives within the previous four weeks, cigarette smoking, and substance abuse or addiction. No other medications and nutritional supplements (vitamins, minerals, fiber products, prebiotics, probiotics, synbiotics, or isoflavones) were allowed during the four weeks prior to study initiation. Details of the study were explained to all subjects and signed informed consent was obtained from all subjects prior to study participation. Withdrawal criteria from this study were adverse drug reactions during the study, inability to comply with the study protocol, and voluntary withdrawal from the study.

### 2.3. Soy and Ciprofloxacin Preparation

The soy-based product used in this study was the UHT soy milk (V-soy®, lot number 8851028004127, expiration date 27 March 2019, manufactured by Green Spot Co., Ltd., Bangkok, Thailand). The mean isoflavone contents of daidzin (the *β*-glycoside form of daidzein) and genistin (the *β*-glycoside form of genistein) were 86.58±0.65 and 47.57±0.36 mg/375 mL, respectively. The amounts of daidzein and genistein were negligible. The ciprofloxacin oral tablets used were CIPROBAY® (ciprofloxacin HCL, batch number BXHSB11, expiration date May 2021, manufactured by Bayer Healthcare Pharmaceuticals Inc., Germany).

### 2.4. Dosage and Administration

**Figure 1:**
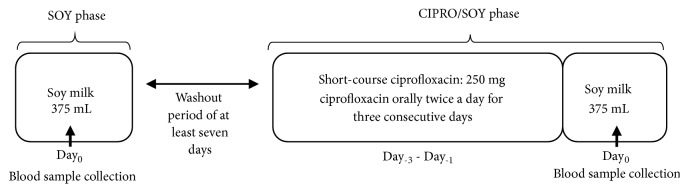
Schedule of administration of soy milk and ciprofloxacin in both phases of the study. The SOY phase represents a single oral administration of soy milk. The CIPRO/SOY phase represents an oral administration of short-course ciprofloxacin followed by a single oral administration of soy milk.

The schedule of administration of soy milk and ciprofloxacin is shown in Figure 1. Each subject was assigned to receive a single oral dose of 375 mL soy milk the morning after an overnight fast of at least eight hours (Day_0_ of the SOY phase). Subjects fasted for an additional two hours after oral administration of the soy milk. Water and lunch were served two and four hours after dosing, respectively. During the first four hours after dosing, subjects were instructed to remain upright. Blood samples were collected at various time points (see below). After a washout period of at least seven days, subjects were assigned to consume 250 mg ciprofloxacin twice a day for three consecutive days (Day_−3_–Day_−1_), followed by a single oral dose of 375 mL soy milk the morning of the following day (Day_0_ of the CIPRO/SOY phase). Administration of soy milk and collection of blood samples were performed in the same manner as in the SOY phase. Identical isoflavone-free foods and beverages were served during the two phases of the study. Subjects were instructed that the ciprofloxacin should not be taken with milk, yogurt, calcium-fortified juice, caffeine, or food or drink containing high levels of magnesium, aluminum, iron, or zinc. Subjects were required to refrain from soy-rich products, e.g., soy milk and tofu, as well as caffeine- and alcohol-containing beverages throughout the study period.

### 2.5. Collection of Blood Samples

For both phases of the study, blood samples were obtained from the forearm by venipuncture through an indwelling intravenous catheter. A 5 mL blood sample for quantification of plasma isoflavones was collected immediately prior to and at 0.5, 1, 2, 4, 6, 8, 10, 12, 24, and 32 hours after oral administration of the soy milk. The blood samples were centrifuged within 30 minutes of collection to separate the plasma and were then kept at −70°C until analysis.

### 2.6. Determination of Plasma Concentrations of Isoflavones

#### 2.6.1. Sample Preparation

Since absorbable aglycones are extensively further metabolized in the intestines and/or liver and are consequently present in the systemic circulation as their *β*-glucuronide and sulfate conjugates [17, 18], the plasma samples were treated with a mixture of *β*-glucuronidases/sulfatase in order to cleave the glucuronide, and sulfate conjugates to their respective aglycones prior to determination of isoflavone concentration. Thus plasma levels of isoflavones reported here are the respective aglycone concentrations. This study focused on the determination of daidzein and genistein because these aglycones and their respective glycosides are known to be the major constituents (>90%) of soy isoflavones [13].

**Figure 2:**
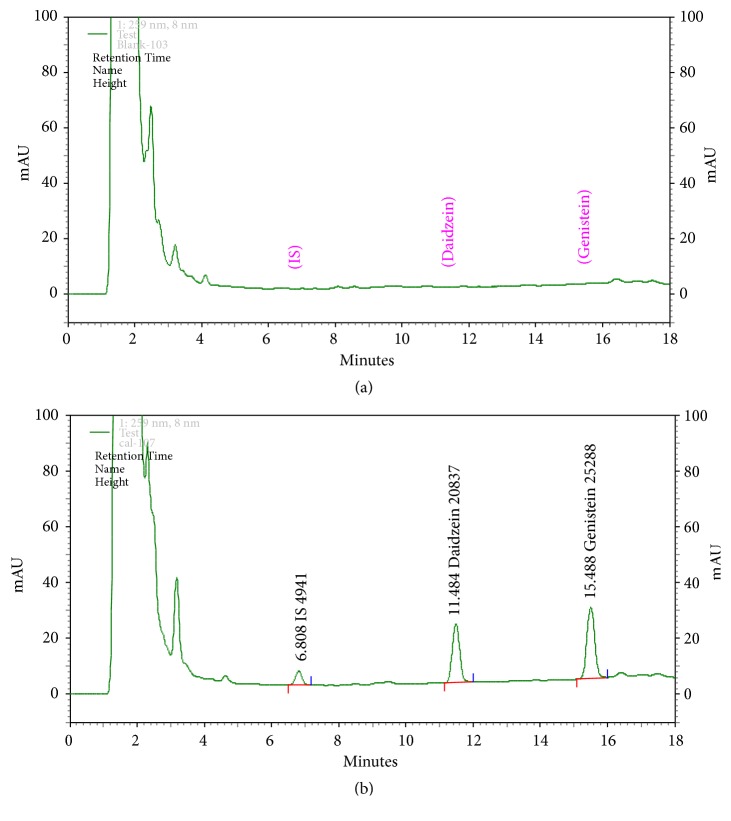
(a) Chromatogram of isoflavone-free plasma. (b) Chromatogram of plasma sample containing daidzein (*k* = 11.484 min) and genistein (*k* = 15.488 min) as well as internal standard (IS,* k* = 6.808 min).

Sample preparation and the method of determination of isoflavone concentrations in plasma were modified from the method described by Teekachunhatean et al. [18, 24–27]. Briefly, a 250 *μ*L aliquot of plasma was treated with 0.15 mL of a mixture of *β*-glucuronidases:sulfatase (97600:2380 units/mL) from* Helix pomatia*, to hydrolyze the glucuronide and sulfate conjugates to aglycones. The enzyme mixture was composed of 0.01 g ethylenediaminetetraacetic acid (EDTA) and 0.1 g ascorbic acid in 10 mL of 0.1 M sodium acetate buffer mixed with 250 *μ*L of* Helix pomatia*. Plasma samples containing the enzyme mixture were heated in a water bath at 37°C for 15 hours and then cooled at room temperature. After enzymatic hydrolysis, plasma samples were spiked with 10 *μ*L of an internal standard (50,000 ng/mL fluorescein in 80% methanol). Fluorescein is recommended as an internal standard in order to correct for unknown losses during the High-Performance Liquid Chromatography (HPLC) analyses of phytoestrogens [28]. It is eluted separately from other UV-absorbing compounds extracted from the soy-based foods and its HPLC retention index is distinct from those of any other soy-based components [29, 30]. After adding an internal standard, plasma samples were deproteinated by adding 1,000 *μ*L of 1% acetic acid in acetonitrile and mixing on a vortex mixer for 30 seconds. The mixture was then centrifuged at 14,000 rpm for ten minutes. An aliquot of the supernatant was isolated and vacuum-evaporated to dryness for three hours at 60°C. The residue was dissolved in 50 *μ*L of mobile phase B (see below), and 5 *μ*L of the sample was injected into the HPLC system. The chromatogram of isoflavone-free plasma and the chromatogram of the plasma sample containing daidzein, genistein, and the internal standard are shown in Figure 2.

#### 2.6.2. High-Performance Liquid Chromatography (HPLC) Conditions

The assay of isoflavones was modified from the HPLC methods and conditions described by Teekachunhatean et al. [18, 24–27]. The chromatographic system consisted of a 5 *μ*m C-18 column equipped with a guard column of the same material. The chromatographic conditions comprised mobile phases A and B. The proportions of 60 mM ammonium acetate in deionized water/acetonitrile/methanol in mobile phases A and B were 250/50/50 and 250/250/220 (v/v/v), respectively. Both mobile phases contained 30 *μ*L of perchloric acid and 250 *μ*L of 1.44 g sodium dodecyl sulfate. Separation was performed at 25°C. A gradient elution of 85% A with 15% B for 12.80 minutes, 33:67 with A:B for 12.81-20.00 minutes, and 85:15 for 20.01-24.00 minutes was scheduled. The mobile phase was maintained at the flow rate of 1 mL/min, and the analyses were detected by UV absorption at 259 nm.

#### 2.6.3. Method Validation

Method validation of isoflavones was performed according to the Food and Drug Administration Guidance for Industry Bioanalytical Method Validation (2018) [31]. The isoflavone content of the samples was determined using a calibration curve of the peak height ratios of the isoflavones and the internal standard versus their respective isoflavone concentrations (37.5, 75, 150, 300, 600, 1,200, and 2,400 ng/mL). Linear regression analysis of peak height ratios of isoflavones versus isoflavone concentrations consistently yielded coefficients of the determinant (r^2^) of 0.999 or better.

The precision of the HPLC method for assay of isoflavones in plasma is reported as the percentage of coefficient of variation (%CV) which was calculated as follows (where SD is the standard deviation and $\overset{-}{X}$ is the mean plasma concentration of the measured isoflavone).

(2) $$\begin{array}{c} \%CV=\frac{SD}{\overset{-}{X}}\times 100 \end{array}$$

Accuracy for the assay of isoflavones in plasma was calculated using the following equation:

(3) $$\begin{array}{c} \%Accuracy=\frac{Measured\;\;concentration}{Spiked\;\;concentraion}\times 100 \end{array}$$

The lower limit of quantification (LLOQ) was defined as the lowest concentration on the calibration curve (37.5 ng/mL) that could be measured with acceptable precision (%CV less than 20%) and acceptable accuracy (80-120%). The LLOQ was determined by analyzing a series of five replicate samples of gradually decreasing concentrations until the lowest concentration with acceptable precision and accuracy was obtained. The mean LLOQ of daidzein and genistein were 36.67±1.20 and 37.19±0.75, respectively. The %CV and %accuracy of LLOQ for daidzein were 3.25% and 98.59%, respectively, whereas, those for genistein were 2.01% and 99.17%, respectively.

Recovery was determined by comparing the peak height of the isoflavone standard samples in the mobile phase, with the peak height of isoflavones in plasma extracted from five sets of three different concentrations of quality control samples (110, 1,100, and 2,200 ng/mL). The extraction recovery of daidzein and genistein in human plasma is shown in Table 1.

For within-day precision, five samples from each of three quality control (QC) samples (110, 1,100, and 2,200 ng/mL) were evaluated with a single calibration curve. For between-day precision, five sets of three different concentrations of QC samples (110, 1,100, and 2,200 ng/mL) were studied on five different days with five concurrent calibration curves. The precision and deviation for assay of daidzein and genistein in human plasma are shown in Table 2.

Short-term stability was tested by preparing quality control samples at two different concentrations (110 and 2,200 ng/mL) in triplicate and analyzing them after remaining on the bench for eight hours at room temperature. For long-term stability evaluation, the quality control samples were stored in a freezer for three months at −70°C for comparison with freshly prepared samples. Freeze-thaw stability was assessed before storage at −70°C and again after three freeze-thaw cycles. Quality control samples after extraction with the same concentration levels in five replicates stored in an autosampler were used to evaluate postpreparative stability. The stability for assay of daidzein and genistein in human plasma is shown in Table 3.

### 2.7. Data Analysis and Statistical Methods

#### 2.7.1. Pharmacokinetic Parameters

The parameters of interest were maximal plasma concentration (C_max_), time to reach peak concentration (T_max_), the area under the plasma concentration-time curve from time zero to the last measurable concentration (AUC_0-t_) and from time zero to infinity (AUC_0-*∞*_), and the terminal half-life (t_1/2_). The individual plasma concentration-time curves were analyzed with a noncompartmental approach using the TopFit pharmacokinetic data analysis program. C_max_ and T_max_ were obtained directly from each subject's plasma concentration-time curve. The terminal elimination rate constant (k_e_) was estimated by log-linear regression of the concentration observed during the terminal phase of elimination. t_1/2_ was calculated as 0.693/k_e_. The AUC_0-t_ was calculated by the trapezoidal rule. The extrapolated AUC_t-*∞*_ was determined as C_t_/k_e_. Total AUC_0-*∞*_ was the sum of AUC_0-t_ + AUC_t-*∞*_.

#### 2.7.2. Statistical Analysis

Pharmacokinetic parameters are presented as mean±SD and median (interquartile range). The mean values of pharmacokinetic parameters obtained from the CIPRO/SOY phase were compared to those of the SOY phase using the paired t-test, whereas the differences between median values of both phases were compared using Wilcoxon's signed rank test. A *p* value of <0.05 was considered statistically significant.

## 3. Results

Eleven postmenopausal subjects completed the study. Their mean age, weight, height, BMI, and serum FSH concentration were 60.18±7.57 years, 53.15±4.50 kg, 1.53±0.04 m, 22.63±1.81 kg/m^2^, and 72.07±16.20 IU/L, respectively (Table 4).

**Figure 3:**
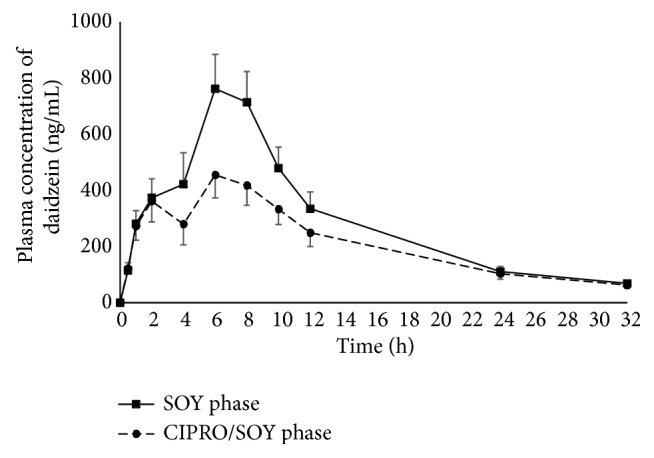
Mean plasma daidzein concentration-time curves from 11 subjects receiving a single oral administration of soy milk (SOY phase) and a short-course oral administration of ciprofloxacin followed by a single oral administration of soy milk (CIPRO/SOY phase). Error bars represent standard error of the mean (SEM).

**Figure 4:**
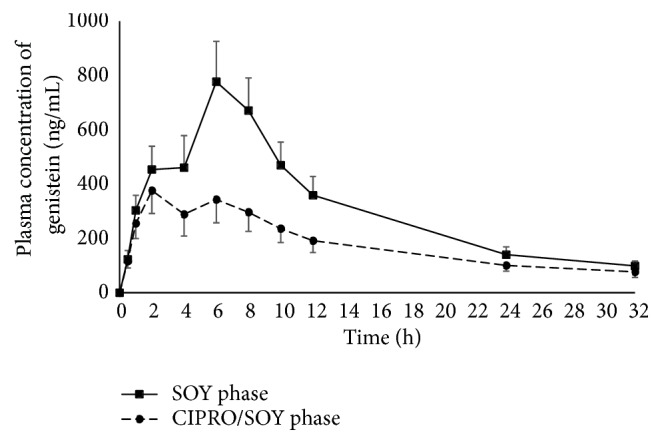
Mean plasma genistein concentration-time curves from 11 subjects receiving a single oral administration of soy milk (SOY phase) and a short-course oral administration of ciprofloxacin followed by a single oral administration of soy milk (CIPRO/SOY phase). Error bars represent standard error of the mean (SEM).

The mean plasma concentration-time curves of daidzein and genistein from the 11 subjects who underwent the SOY and CIPRO/SOY phases are shown in Figures 3 and 4, respectively. The mean plasma concentration-time curves of both aglycones in both phases exhibited a biphasic pattern.

In the SOY phase, the first and second peak concentrations were attained at approximately two to four hours and at six hours, respectively. The second peak concentration of each aglycone was markedly higher than the first peak. It is noteworthy that the second peaks of both aglycones obtained from the CIPRO/SOY phase were remarkably lower than those of the SOY phase, whereas the short-course oral ciprofloxacin given in the CIPRO/SOY phase caused a slight reduction in the first peak concentrations of both aglycones in comparison to those observed in the SOY phase.

The pharmacokinetic parameters of daidzein and genistein (C_max_, T_max_, AUC_0-t_, AUC_0-*∞*_, and t_1/2_) after oral administration of soy milk obtained from both phases are shown in Tables 5 and 6. The mean/median values of C_max_, AUC_0-t_, and AUC_0-*∞*_ of both daidzein and genistein, as well as the T_max_ of genistein obtained from the CIPRO/SOY phase, were significantly lower than those of the SOY phase. However, the mean/median values of T_max_ of daidzein, as well as t_1/2_ of both daidzein and genistein, did not differ significantly between the two phases.

## 4. Discussion

In this one-group pre-post treatment study, the dependent variables of interest (pharmacokinetic parameters of isoflavones) were measured in a single group of 11 participants and then measured again in the same group after exposure to an intervention (a short-course of oral ciprofloxacin) to determine the difference between the initial (pretreatment, SOY phase) and second (posttreatment, CIPRO/SOY phase) measurements. With this within-subjects design, the conditions in pre- and posttreatment phases were assumed to be equivalent with regard to individual difference variables because the same participants participated in both conditions. In addition, a washout period between the SOY and the CIPRO/SOY phases of at least seven days is considered sufficient to avoid carryover effects as that period is >4-5 times t_1/2_ of isoflavones, ensuring that any isoflavones absorbed during the SOY phase were entirely eliminated before initiation of the CIPRO/SOY phase. As it is difficult to estimate carryover effects on the restoration of gut microbiota after taking antibiotics, this study was conducted in a fixed sequence: the SOY phase followed by the CIPRO/SOY phase.

The biphasic pattern of plasma concentration-time curves of the isoflavones obtained from the SOY phase was consistent with findings previously reported in studies of postmenopausal Thai women [18, 24–27]. The first and second peaks are due to absorption of isoflavones in the small and the large intestine, respectively [32]. In the upper small intestine, conversion of isoflavone glycosides (the predominant forms in soy milk) to aglycones facilitates rapid absorption via passive diffusion across the intestinal brush border [33]. This conversion involves the action of intestinal lactase phlorizin hydrolase [34], enterocytic *β*-glucosidase [35], and microbial *β*-glucosidases [36, 37]. The remaining isoflavone glycosides that are not absorbed in the upper intestinal tract would pass through to the lower intestinal tract, where gut microbial *β*-glycosidases further cleave them into aglycones prior to absorption [38]. Additionally, the metabolites of isoflavones (glucuronide and sulfate conjugates), which are excreted into the intestinal tract via the biliary tract, can be deconjugated by gut microbial *β*-glycosidases and can undergo enterohepatic recycling. This phenomenon also contributes to the appearance of a second peak [39]. Since the lower intestinal tract (especially the colon) is the major site of isoflavone absorption [32], it was not surprising that the second peak concentration of each aglycone obtained from the SOY phase was markedly higher than the first peak. There are various types of microbiota involved in the conversion of isoflavone glycosides to readily absorbable aglycones, e.g., streptococcus, lactobacillus, bifidobacterium, bacteroides, enterobacteria, eubacteria, and enterococcus [3, 38, 40].

Although ciprofloxacin (250 mg, orally, twice daily for three consecutive days) is considered to be an alternative or second-line antimicrobial agent for treatment of acute uncomplicated cystitis in otherwise healthy women according to international guidelines on urological infections [20], this regimen is commonly used in medical practice in Thailand due to the high prevalence of pathogens resistant to first-line regimens. This regimen may also be indicated when beta-lactam agents are contraindicated. Investigation of the impact of a three-day regimen on gut microbiota ecology has found a significant reduction in enterobacteria and a slight increase in the amount of bifidobacteria and anaerobic cocci [41]; furthermore, a higher dose (500 mg orally twice daily for five consecutive days) has been reported to cause a reduction in the amount of enterobacteria and enterococci [42]. The potential impact of ciprofloxacin on gut microbiota ecology could possibly affect the oral bioavailability of ingested isoflavone glycosides.

In this study, short-course oral ciprofloxacin caused a marked decrease in the second peak concentrations, but only a slight decrease in the first peak concentrations of both aglycones. These findings presumably reflect the impact of ciprofloxacin on the quantities of microbiota which are responsible for the conversion of isoflavone glycosides to readily absorbable aglycones. Since the microbiota plays a crucial role in the absorption of isoflavone glycosides in the lower intestinal tract but partly contributes to an absorption in the upper small intestine, it was not surprising that oral ciprofloxacin caused a greater impact on the second peak than the first peak observed in the CIPRO/SOY phase. The findings of this study are consistent with previous studies demonstrating that use of oral antibiotics (erythromycin and neomycin) together with mechanical bowel preparation (in order to radically reduce gut microbiota) negatively affect the second peak [32]. On the other hand, either prebiotic or synbiotic supplementation (which can facilitate the growth of gut microbiota) results in an enhancement of the second peak in postmenopausal women [25, 26].

The presumed decrease in the amount of gut microbiota appears to be the likely cause of the significant reduction in the mean/median values of C_max_, AUC_0-t_, and AUC_0-*∞*_ of both daidzein and genistein obtained from the CIPRO/SOY phase compared to those of the SOY phase. With T_max_ obtained from the CIPRO/SOY phase, ciprofloxacin reduced the second peak of genistein concentration below that of the first peak, significantly shifting T_max_ of genistein toward the first peak. In contrast, the second peak of daidzein concentration was not lower than the first peak. As a result, T_max_ of daidzein was not altered from that observed in the SOY phase. In addition, t_1/2_ of both daidzein and genistein did not differ significantly between the two phases, suggesting that ciprofloxacin has a negligible impact on isoflavone elimination.

The findings of this study suggest that a three-day oral ciprofloxacin reduces the oral bioavailability of isoflavones (as evidenced by the decrease in AUC and C_max_) following ingestion of soy milk in postmenopausal women. This food-drug interaction appears to be of clinical relevance in cases where this ciprofloxacin regimen is prescribed to treat illnesses, e.g., acute uncomplicated cystitis, in postmenopausal women receiving oral isoflavones (especially glycoside forms) or nonfermented soy-based products. However, it is still unclear how long the carryover effect of ciprofloxacin on isoflavone pharmacokinetic lasts after treatment discontinuation. A previous study suggested that gut microbiota ecology might return to normal within two weeks [42]. Further studies addressing this issue are required.

Some limitations of this study should be addressed. First, there is a diversity of gut microbiota ecology among human populations [43–46]. This study was conducted in Thai postmenopausal women; the findings could be different in other racial and ethnic groups as well as other geographic locations. Second, the 250 mg of ciprofloxacin in this study was given twice daily for three consecutive days; the effect of ciprofloxacin might differ with a different dose and/or duration. Third, the effect of food-drug interaction also would probably differ if other fluoroquinolones and soy-based products were coadministered. Finally, the lack of direct quantification of fecal microbiota to determine the impact of oral ciprofloxacin on gut microbiota ecology was also considered a limitation.

## 5. Conclusions

A three-day regimen of oral ciprofloxacin followed by a single oral administration of soy milk causes a significant decrease in C_max_, AUC_0-t_, and AUC_0-*∞*_ of both daidzein and genistein, as well as T_max_ of genistein compared to a single oral dose of soy beverage alone. However, T_max_ of daidzein and t_1/2_ of aglycones, genistein, and daidzein, are not significantly different.

## Figures and Tables

**Table 1:** Extraction recovery of daidzein and genistein in human plasma (n=5).

Compound	Concentration (ng/mL)	Peak height (mAU)		%Recovery
		In mobile phase	In plasma	
		(mean±SD)	(mean±SD)	
Daidzein	110	1573±11.39	1327±43.33	84.38
	1100	14531±95.16	12371±233.50	85.14
	2200	27001±498.58	24130±1453.77	89.36
Average recovery				86.29

Genistein	110	2176±49.60	1962±137.96	90.17
	1100	19789±131.30	16789±287.45	84.84
	2200	34279±634.20	31042±1910.87	90.56
Average recovery				88.52

**Table 2:** Precision and accuracy for assay of daidzein and genistein in human plasma.

Compound	Concentration (ng/mL)	Within-day (n=5)			Between-day (n=5)		
		Measured concentration (ng/mL), mean±SD	Precision (%CV)	Accuracy (%)	Measured concentration (ng/mL), mean±SD	Precision (%CV)	Accuracy (%)
Daidzein	110	105.54±1.20	1.14	95.95	112.01±4.41	3.94	101.83
	1100	1169.95±13.06	1.12	106.36	1106.44±57.40	5.19	100.59
	2200	2271.59±24.85	1.09	103.25	2267.39±120.66	5.32	103.06
Average			1.12	101.85		4.81	101.82

Genistein	110	108.79±1.99	1.83	98.90	113.16±8.18	7.23	102.87
	1100	1131.92±12.52	1.11	102.90	1070.99±76.98	7.19	97.36
	2200	2203.29±26.97	1.22	100.15	2199.30±125.02	5.68	99.97
Average			1.39	100.65		6.70	100.07

**Table 3:** Stability for assay of daidzein and genistein in human plasma.

Compound	Concentration (ng/mL)	Short-term stability (8 hours, n=3) %Remaining	Long-term stability (3 months, n=3) %Remaining	Freeze-thaw stability (n=3) %Remaining	Post-preparative stability (n=5) %Remaining
Daidzein	110	98.42	105.19	86.26	98.01
	2200	98.54	94.57	100.14	98.11
Average stability		98.48	99.88	93.20	98.06

Genistein	110	99.56	104.44	91.15	96.76
	2200	99.25	95.67	110.88	96.94
Average stability		99.40	100.05	101.02	96.85

**Table 4:** Demographic characteristics of the 11 subjects participating in this study.

Subject	Age	Weight	Height	BMI	FSH
No.	(y)	(kg)	(m)	(kg/m^2^)	(IU/L)
1	69	56.00	1.50	24.89	63.99
2	54	51.00	1.52	22.07	85.14
3	54	49.70	1.58	19.91	56.78
4	59	50.50	1.58	20.36	98.80
5	51	56.00	1.52	24.24	53.76
6	62	52.00	1.48	23.74	80.66
7	63	52.00	1.52	22.66	70.78
8	59	60.00	1.58	24.03	82.08
9	69	47.00	1.48	21.46	48.76
10	72	61.00	1.57	24.75	62.73
11	50	49.50	1.54	20.87	89.33

Mean	60.18	53.15	1.53	22.63	72.07

SD	7.57	4.50	0.04	1.81	16.20

**Table 5:** Pharmacokinetic parameters of daidzein obtained from 11 subjects receiving a single oral administration of soy milk (SOY Phase) and a short-course oral ciprofloxacin followed by a single oral administration of soy milk (CIPRO/SOY phase).

Pharmacokinetic parameters	Daidzein			
	SOY phase		CIPRO/SOY phase	
	Mean±SD	Median (IQR)	Mean±SD	Median (IQR)
C_max_ (ng/mL)	833.12±351.17	752.60 (298.52)	511.17±243.74^*∗*^	497.19 (323.01)^*∗∗*^
T_max_ (h)	6.36±1.21	6.00 (1.00)	6.55±1.29	6.00 (2.00)
AUC_0-t_ (ng.h/mL)	8553.84±5424.86	6826.87 (5759.28)	5977.10±4256.78^*∗*^	5599.80 (4391.67)^*∗∗*^
AUC_0-*∞*_ (ng.h/mL)	9431.94±5411.69	7254.24 (4777.05)	6746.22±4403.37^*∗*^	6003.36 (4164.71)^*∗∗*^
t_1/2_ (h)	6.52±1.89	6.23 (1.64)	6.42±2.07	6.36 (1.45)

Data represent mean±SD and median (interquartile range, IQR). ^*∗*^*p* <0.05 versus SOY phase using a paired t-test. ^*∗∗*^*p* <0.05 versus SOY phase using Wilcoxon's signed-rank test.

**Table 6:** Pharmacokinetic parameters of genistein obtained from 11 subjects receiving a single oral administration of soy milk (SOY Phase) and a short-course oral ciprofloxacin followed by a single oral administration of soy milk (CIPRO/SOY phase).

Pharmacokinetic parameters	Genistein			
	SOY phase		CIPRO/SOY phase	
	Mean±SD	Median (IQR)	Mean±SD	Median (IQR)
C_max_ (ng/mL)	826.64±462.97	731.95 (521.01)	434.75±276.24^*∗*^	306.17 (214.37)^*∗∗*^
T_max_ (h)	6.00±1.55	6.00 (0.00)	3.27±2.24^*∗*^	2.00 (2.00)^*∗∗*^
AUC_0-t_ (ng.h/mL)	9583.48±6482.03	8911.53 (7711.23)	4956.98±4263.76^*∗*^	3834.93 (4106.07)^*∗∗*^
AUC_0-*∞*_ (ng.h/mL)	10540.60±7024.65	9284.07 (7789.07)	5791.53±4567.84^*∗*^	4366.76 (3976.55)^*∗∗*^
t_1/2_ (h)	7.40±1.20	7.26 (1.57)	7.54±1.69	7.16 (1.69)

Data represent mean±SD and median (interquartile range, IQR). ^*∗*^*p* <0.05 versus SOY phase using a paired t-test. ^*∗∗*^*p* <0.05 versus SOY phase using Wilcoxon's signed-rank test.

## Data Availability

The data used to support the findings of this study are available from the corresponding author upon request.

## Abbreviations

AUC_0-t_: Area under the plasma concentration-time curve from time zero to the last measurable concentration
AUC_0-*∞*_: Area under the plasma concentration-time curve from time zero to infinity
C_max_: Maximal plasma concentration
k_e_: Terminal elimination rate constant
T_max_: Time to reach peak concentration
t_1/2_: Terminal half-life.
